# Supplementary material for: Polyketide synthase gene clusters in the ericoid mycorrhizal fungus Oidiodendron maius: genome-wide prediction, expression profiling, and functional characterization of knockout mutants
Source: Front Plant Sci. 2026 Sep 16;17:1924430. doi: 10.3389/fpls.2026.1924430 (PMC13623903; doi:10.3389/fpls.2026.1924430)
Supplement: Supplementary file 1 [file SupplementaryFile1.zip › Supplementary Materials/Supplementary Folder 1.zip!/Oidma1_AssemblyScaffolds_Repeatmasked/knownclusterblast/cluster47/jgi.p_Oidma1_136193_mibig_hits.html]

| MIBiG Protein | Description | MIBiG Cluster | MiBiG Product | % ID | % Coverage | BLAST Score | E-value |
| --- | --- | --- | --- | --- | --- | --- | --- |
| AMR44277.1 | putative\_salicylate\_hydroxylase | BGC0001398\_c4 | Phomopsins\_biosynthetic\_gene\_cluster | 77 | 98.7080103359 | 598 | 6.1e-171 |
| AAT65710.1 | putative\_monooxygenase | BGC0000629 | Aflatrem\_biosynthetic\_gene\_cluster | 31 | 103.359173127 | 179 | 6.8e-45 |
| AAS92536.1 | HDX1 | BGC0001044 | Sirodesmin\_biosynthetic\_gene\_cluster | 31 | 94.0568475452 | 139 | 6e-33 |
| EAQ84784.1 | hypothetical\_protein | BGC0001219 | Shanorellin\_biosynthetic\_gene\_cluster | 31 | 97.9328165375 | 136 | 5.1e-32 |
| EWM63055.1 | monooxygenase | BGC0000679 | Diazepinomicin\_biosynthetic\_gene\_cluster | 29 | 106.718346253 | 124 | 2.6e-28 |
| ADD82995.1 | PtnB3 | BGC0001156 | Platencin\_biosynthetic\_gene\_cluster | 27 | 83.9793281654 | 84 | 3.9e-16 |
| EAA65601.1 | hypothetical\_protein | BGC0000022 | Asperfuranone\_biosynthetic\_gene\_cluster | 26 | 99.7416020672 | 64 | 2.4e-10 |
| CBF90103.1 | conserved\_hypothetical\_protein | BGC0001271 | Emericellin\_biosynthetic\_gene\_cluster | 22 | 99.7416020672 | 61 | 2.1e-09 |
